# Has the development of artificial intelligence promoted urban pollutant and carbon emission reduction? Evidence from China

**DOI:** 10.3389/fpubh.2025.1739342

**Published:** 2026-01-13

**Authors:** Mengyu Li, Tangfa Liu, Guancheng Wu, Ji Lin, Bingnan Guo

**Affiliations:** 1 School of Humanities and Social Sciences, Jiangsu University of Science and Technology, Zhenjiang, China; 2 School of Economics and Management, Gannan University of Science and Technology, Ganzhou, China; 3 School of Business, Durham University, Durham, United Kingdom; 4 School of Economics and Management, Wenzhou University of Technology, Wenzhou, China

**Keywords:** artificial intelligence, double machine learning, green technology innovation, green total factor energy efficiency, industrial structure, pollutant and carbon emission reduction

## Abstract

Driven by the “dual carbon” goals, China’s economy is gradually advancing toward green transformation, and leveraging new-generation information technologies to facilitate environmental governance has become a key national strategic priority. Based on the panel data of 282 prefecture-level cities in China from 2009 to 2023, this paper adopts a double machine learning model to systematically investigate the impact of artificial intelligence (AI) on urban pollutant and carbon emission reduction, as well as its underlying mechanisms and regional heterogeneity. The results show that AI significantly promotes urban pollutant and carbon emission reduction, with the core regression coefficient being −0.026. Mechanism analysis reveals that AI exerts its emission reduction effect through three channels: improving green total factor energy efficiency, optimizing industrial structure, and driving green technology innovation. The conclusion remains robust after a series of tests, including excluding municipalities directly under the central government, winsorizing outliers, and resetting the double machine learning model. Heterogeneity analysis indicates that the emission reduction effect of AI is prominent in the Beijing-Tianjin-Hebei urban agglomeration, Chengdu-Chongqing urban agglomeration, as well as the northern coastal, middle Yellow River, southwest and northwest regions of China, while the effect is not significant in the Yangtze River Delta, Pearl River Delta and other regions due to industrial structure constraints and uneven policy implementation. This study verifies the causal effect of AI on urban pollutant and carbon emission reduction at the micro-city level, expands the application boundary of double machine learning in the field of environmental economics, and provides targeted empirical evidence for formulating differentiated “AI for dual carbon initiative” policies in different regions, thus offering important theoretical support and practical reference for advancing the green and low-carbon transformation of China’s economy.

## 1 Introduction

Since the beginning of the 21st century, global environmental issues have become increasingly prominent, particularly the intensification of greenhouse gas emissions and climate change, which constitute significant challenges faced by various countries (1, 2). Against this backdrop, pollutant and carbon emission reduction have become essential strategies for promoting sustainable economic development and addressing climate change (3). Since the reform and opening up, China’s economy has experienced remarkable growth, with its GDP ranking second in the world. The process of industrialization has deepened, establishing the most complete industrial system globally. However, the high-energy consumption and elevated emissions paths under the traditional industrialization model have also posed severe challenges for environmental governance—regional smog occurs frequently, and carbon emissions remain high (4, 5). In 2023, China’s carbon dioxide emissions reached 12.6 billion tons, accounting for approximately 34% of the global total emissions. The pressure on the ecological environment continues to increase, particularly concerning the energy structure. In 2023, China’s total energy consumption reached 5.72 billion tons of standard coal, with fossil fuels such as coal and oil still accounting for over 80%. The pollution emissions resulting from the combustion of these fuels are in stark contrast to the promotion of the “dual carbon” goals (6). Therefore, solely relying on traditional environmental governance methods has become insufficient to break through the bottleneck in emission reduction. It is necessary to depend on technological innovation to reconstruct production processes and promote a comprehensive green transformation of the economy and society (7).

Pollutant and carbon emission reduction are vital initiatives for promoting a comprehensive green transformation of economic and social development in China’s new development stage. China has made significant contributions to addressing climate change and reducing environmental pollution, actively implementing low-carbon policies and encouraging a transition in its economic development model (8). However, under the “coal-rich, oil-poor, and gas-poor” energy system, as China intensifies its pollution control efforts, the pressure for end-of-pipe emission reductions has increasingly intensified, presenting severe challenges to the synergy between pollutant and carbon emission reduction (9). Cities, as the most important spatial hubs of modern economic development, are rapidly aggregating production factors such as resources, labor, capital, and technology. The industrial sector in cities has developed rapidly, generating significant carbon emissions and pollutants, which positions cities as the primary battleground for pollutant and carbon emission reduction.

Technological advancement is widely recognized as an essential strategy for reducing environmental pollution and carbon emissions. In recent years, with the advancement of the latest technological revolution, there has been an exponential growth of new-generation information technologies globally, such as 5G, big data, and cloud computing. Of these emerging technologies, the surge of AI has been exceptionally strong and is exerting a profound impact on economic growth (10, 11), employment (12, 13), wealth distribution (14), industrial structure (15), international trade (16, 17), as well as various impacts on microeconomic agents such as companies and citizens (18, 19). AI boasts the technical characteristics of real-time monitoring, precise regulation, and intelligent decision-making, which are highly compatible with the refined governance needs of pollutant and carbon emission reduction. This endows AI with broader prospects for application in addressing environmental issues.

China places great emphasis on advancing artificial intelligence development and aims to strategically seize this opportunity to establish a leading position in the field. In 2017, China released the “New Generation Artificial Intelligence Development Plan,” implementing a comprehensive strategy at the national level. In 2022, the Chinese government report proposed fostering new growth drivers through innovative information technologies, artificial intelligence, biotechnology, renewable energy, innovative materials, advanced machinery, and environmentally friendly practices. In this context, artificial intelligence plays a pivotal role in the current technological revolution and industrial transformation, facilitating the green transition of production methods. It promotes this transition by advancing clean production technologies, continuous monitoring, and precise control of pollution emissions (20, 21). Therefore, can the development of artificial intelligence achieve synergistic effects in pollutant and carbon emission reduction in Chinese cities? If so, what are the underlying mechanisms? What differences in urban characteristics and spatial configurations of artificial intelligence applications influence the synergy of pollutant and carbon emission reduction? To address these questions, we utilize panel data from Chinese cities between 2009 and 2023 as a research sample and employ a dual machine learning model to evaluate the impact of artificial intelligence development on urban pollutant and carbon emission reduction. Additionally, we adopt a theoretical model decomposition to study its pathways. The study finds that the development of artificial intelligence promotes urban pollutant and carbon emission reduction through three pathways: improving energy efficiency, upgrading industrial structure, and fostering green technological innovation, which holds significant practical implications.

Compared to existing literature, this paper’s incremental contributions are primarily reflected in the following aspects: ① Existing studies mostly adopt provincial or national-level data as the analysis unit, while this study focuses on the micro-scale of prefecture-level cities and conducts an in-depth analysis of heterogeneity at the urban agglomeration level. It specifically reveals the significant differences in AI’s emission reduction effects between urban agglomerations such as the Beijing-Tianjin-Hebei and Chengdu-Chongqing, and those like the Yangtze River Delta and Pearl River Delta. This fills the gap in the refined research on emission reduction heterogeneity at the urban agglomeration dimension; ② While some existing literature only identifies a single or two types of emission reduction mechanisms, this study systematically verifies three core transmission paths: green total factor energy efficiency, industrial structure optimization, and green technology innovation. It also quantifies the mediating effect coefficient of each mechanism, which deepens the understanding of the action paths of AI in emission reduction; ③ Compared with the traditional regression methods adopted in existing studies, this study employs the double machine learning model, which effectively addresses the challenges of high-dimensional variable interference and the identification of nonlinear relationships between variables, thus improving the reliability and accuracy of causal inference.

## 2 Theoretical framework

### 2.1 The direct effects of artificial intelligence development on synergistic improvement in pollutant and carbon emission reduction

With the deepening advancement of industrial modernization and digital technologies, artificial intelligence has gradually emerged as a pivotal driver for enhancing environmental governance efficiency, with its direct empowering effects on Pollutant and Carbon Emission Reduction becoming increasingly prominent (22). However, the proxy indicator for AI adopted in this paper—industrial robot density—can only reflect the level of automation in the manufacturing sector, while manufacturing is merely a subset of the broad AI ecosystem. Areas such as smart grids, AI-optimized transportation systems, and building management all fall under the category of generalized AI, and AI applications in these fields can also provide innovative solutions for pollutant and carbon emission reduction. Specifically, artificial intelligence can directly reduce pollutants and carbon emissions by optimizing production processes: for example, industrial robots use precise control algorithms to replace manual operations, avoiding energy waste and excessive emissions caused by human operational deviations (23, 24). Additionally, artificial intelligence can enhance emission reduction effects through real-time monitoring and dynamic adjustments. Industrial robots equipped with sensors can collect real-time data on energy consumption and pollutant concentration during production processes, rapidly identifying risks of exceeding emission limits through algorithms, and promptly adjusting production parameters to avoid peaks in pollution emissions, thus mitigating the lag associated with traditional post-event governance. Furthermore, artificial intelligence aids in advancing the precision of energy utilization, directly reducing emissions resulting from fossil fuel consumption. By constructing energy consumption prediction models, industrial robots can plan production schedules in advance, prioritizing high-energy production during clean energy supply periods while simultaneously minimizing ineffective energy consumption caused by idle equipment, thus directly decreasing carbon emissions at the energy usage end.

Therefore, as a new technological element, artificial intelligence reconstructs the energy utilization and pollution control models of industrial production, effectively shifting the boundary of possibilities for pollutant and carbon emission reduction. This makes the reduction effects more evident, positioning artificial intelligence as a key factor in promoting pollutant and carbon emission reduction.

Accordingly, we propose Research Hypothesis 1: The development of artificial intelligence can significantly promote urban pollutant and carbon emission reduction.

### 2.2 The indirect effects of artificial intelligence development on synergistic improvement in pollutant and carbon emission reduction

#### 2.2.1 The mediating effect of green total factor energy efficiency

Artificial intelligence promotes pollutant and carbon emission reduction by enhancing the overall green energy efficiency in cities, where the core logic of this intermediary pathway lies in incorporating energy consumption and pollutant emissions into a comprehensive evaluation framework. This systematically reflects how energy inputs influence economic output and alter environmental costs through a collaborative optimization framework. Improvements in overall green energy efficiency signal the achievement of dual goals: enhancing energy utilization efficiency while reducing pollution emissions, all while ensuring the continuation of economic activities (8, 25). The empowering effect of artificial intelligence on this efficiency is distinctly directed. On one hand, real-time monitoring systems equipped in industrial robots can accurately collect energy consumption and emission data from various locations, leveraging algorithms to identify high-energy-consuming segments at the regional level and promoting the flow of energy resources towards efficient utilization sectors (26, 27). On the other hand, machine learning algorithms can dynamically optimize production sequences and adjust production parameters, thereby eliminating redundant energy consumption and enhancing the overall green energy efficiency of cities.

Meanwhile, a critical question arises: How does enhancing overall green energy efficiency specifically promote pollutant and carbon emission reduction? At the urban level, energy-intensive regions often exhibit significantly higher carbon and pollutant emissions per unit of GDP due to low energy utilization efficiency compared to more efficient regions. When overall green energy efficiency improves, cities can achieve greater economic value with the same energy input, or consume less energy for the same economic output; the former reduces energy intensity per unit of GDP, while the latter directly decreases carbon and pollutant emissions from fossil fuel combustion, collectively driving down the intensity of urban pollution emissions (28). In addition, the enhancement of overall green energy efficiency can alleviate the rigid constraints from energy consumption to pollution emissions, allowing cities to pursue economic growth objectives without relying on energy-intensive and high-emission production modes. This results in a synergistic advancement of economic development and environmental governance. Therefore, combining the empowering role of artificial intelligence development on overall green energy efficiency with the driving effect of this efficiency on pollutant and carbon emission reduction.

We propose Hypothesis 2: The development of artificial intelligence can promote urban pollutant and carbon emission reduction through the enhancement of overall green energy efficiency.

#### 2.2.2 The mediating effect of industrial structure

Industrial structure serves as an important intermediary pathway through which artificial intelligence drives urban pollutant and carbon emission reduction. The core logic lies in the optimization direction of the industrial structure shifting from labor-intensive to technology-intensive, and from a focus on the secondary sector to a collaborative emphasis on the tertiary sector. An increase in the proportion of the tertiary sector can effectively reduce regional pollution emission intensity. Artificial intelligence facilitates the adjustment of industrial structure through the following pathways: First, industrial robots replace jobs in energy-intensive manufacturing, prompting labor to shift towards the low-pollution tertiary sector, thereby directly increasing the share of the tertiary sector. Second, digital transformation optimizes manufacturing production efficiency, phases out outdated energy-intensive capacities, and generates demand for productive services, further promoting the adjustment of the industrial structure towards high technology and low emissions (29).

When the industrial structure is optimized, the low energy consumption characteristics of the tertiary sector will directly lower overall pollution levels in the city. At the same time, the increased share of high-tech manufacturing within the secondary sector also reduces industrial pollution emissions through the optimization of production processes, collectively lowering the intensity of urban pollution emissions. Moreover, the adjustment of industrial structure can alleviate the conflict between economic growth and pollution emissions, allowing cities to pursue economic growth objectives without the need for high-emission production modes (30).

Therefore, we propose Hypothesis 3: The development of artificial intelligence can optimize the industrial structure by promoting the increased share of the tertiary sector, thereby indirectly facilitating urban pollutant and carbon emission reduction.

#### 2.2.3 The mediating effect of green technological innovation

Green technology innovation is the third key intermediary pathway through which artificial intelligence drives urban pollutant and carbon emission reduction, primarily manifested in the upgrades of pollution control technologies and breakthroughs in low-carbon production technologies, as indicated by the volume of green invention patent applications. Such technologies can reduce pollution emission intensity at the source and enhance treatment efficiency, thereby promoting regional environmental governance (8, 31). The driving pathways of artificial intelligence have two facets. First, the energy consumption and emission data accumulated by industrial robots provide empirical evidence for the research and development of green technologies, assisting research institutions in identifying technological bottlenecks (32). Second, machine learning algorithms accelerate the research and development process, shortening the timeframe for green technologies from laboratory experimentation to industrialization, while also broadening the application scenarios of these technologies in energy-intensive industries.

How does green technology innovation facilitate pollutant and carbon emission reduction? High-pollution areas are often constrained by traditional technologies, making it challenging to effectively reduce emissions. In contrast, green technology innovation can overcome these barriers. An enhancement in green technology innovation capacity is often accompanied by an increase in the number of green invention patents and accelerated technology transfer. On one hand, advanced pollution control technologies significantly enhance treatment efficiency and reduce end-of-pipe emissions; on the other hand, low-carbon production technologies can reduce fossil fuel consumption at the source, thereby decreasing carbon emissions. Together, these two factors contribute to the dual reduction of urban pollutant and carbon emission reduction. Furthermore, green technology innovation also elevates the overall technological level of enterprises within the region, thereby amplifying the impact of pollutant and carbon emission reduction (33).

In summary, we propose Hypothesis 4: The development of artificial intelligence promotes green technology innovation by driving an increase in the number of green invention patent applications, thereby indirectly facilitating urban pollutant and carbon emission reduction.

## 3 Empirical analysis

### 3.1 Research design

#### 3.1.1 Model construction

This paper aims to investigate the impact of artificial intelligence development on urban pollutant and carbon emission reduction. Currently, most studies on policy evaluation primarily employ the Difference-in-Differences method, Synthetic Control method, and Regression Discontinuity Design. However, these traditional causal inference models have several limitations in application. For instance, the Difference-in-Differences method requires that the treatment and control groups exhibit the same trend of change, meaning that the study samples must satisfy the parallel trends assumption (8, 34). Although the Synthetic Control method avoids the challenge of finding treatment and control groups with the same trend by constructing a control group, it is primarily applicable in “one-to-many” scenarios. The Regression Discontinuity Design not only has high data requirements but also can only estimate the local average treatment effect near the cutoff point. To address the shortcomings of traditional models, some scholars have combined machine learning with causal inference, with Double Machine Learning serving as a typical representation of this approach (35). Compared to traditional policy evaluation models, Double Machine Learning has unique advantages in variable selection and model estimation: it uses regularization algorithms to automatically select high-dimensional control variables, addressing multicollinearity and the curse of dimensionality that plague traditional models, thus improving predictive accuracy (36, 37). On the other hand, Double Machine Learning models can handle nonlinear relationships between variables, thus preventing errors in model specification. Based on this, this paper employs a Double Machine Learning model to assess the impact of artificial intelligence development on urban pollutant and carbon emission reduction. Specifically, the partially linear Double Machine Learning model is as follows:


$$EI_{\mathrm{it}}=\theta_{0}AI_{\mathrm{it}}+g(X_{\mathrm{it}})+U_{\mathrm{it}}$$ (1)

$$E(U_{\mathrm{it}}|AI_{\mathrm{it}},X_{\mathrm{it}})=0$$ (2)

In this framework, i represents the city, and t denotes the year. $EI_{\mathrm{it}}$ indicates the reverse level of urban pollutant and carbon emission reduction in the city, while $AI_{\mathrm{it}}$ serves as the explanatory variable representing the development of artificial intelligence. The parameter $\;\theta_{0}\;$ denotes the policy effect, which reflects the influence of artificial intelligence development on the promotion of pollutant and carbon emission reduction at the urban level. Additionally, $X_{\mathrm{it}}$ represents potential high-dimensional control variables, and $g(X_{\mathrm{it}})$ illustrates the potential nonlinear relationship between these high-dimensional control variables and urban pollutant and carbon emission reduction. Since this relationship is largely unknown, we estimate its form using machine learning algorithms as $\hat{g}(X_{\mathrm{it}})$ The term $\;U_{\mathrm{it}}\;$ represents the error term, with a conditional mean of zero. When employing $\hat{g}(X_{\mathrm{it}})$ , it is necessary to introduce the Neyman orthogonality concept to construct the following matrix:

From Formula 1, the estimated value of the policy effect ${\hat{\theta }}_{0}\;$ can be derived as follows:


$$\begin{array}{c} {\hat{\theta }}_{0}={\left(\frac{1}{n}\sum_{i\in I,t\in T}A{I_{\mathrm{it}}}^{2}\right)}^{-1}\frac{1}{n}\sum_{i\in I,t\in T}[EI_{\mathrm{it}}-\hat{g}(X_{\mathrm{it}})] \end{array}$$ (3)

Here, n represents the total sample size. Based on the estimated value of the policy effect, the estimation bias of this policy effect can be further examined:


$$\begin{array}{c} \sqrt{n}({\hat{\theta }}_{0}-\theta_{0})={\left(\frac{1}{n}\sum_{i\in I,t\in T}\;A{I_{\mathrm{it}}}^{2}\right)}^{-1}\frac{1}{\sqrt{n}}\sum_{i\in I,t\in T}AI_{\mathrm{it}}U_{\mathrm{it}}\\ +{\left(\frac{1}{n}\sum_{i\in I,t\in T}\;A{I_{\mathrm{it}}}^{2}\right)}^{-1}\frac{1}{\sqrt{n}}\sum_{i\in I,t\in T}\;AI_{\mathrm{it}}[g(X_{\mathrm{it}})-\hat{g}(X_{\mathrm{it}})] \end{array}$$ (4)

Here, ${\left(\frac{1}{n}\sum_{i\in I,t\in T}A{I_{\mathrm{it}}}^{2}\right)}^{-1}\frac{1}{\sqrt{n}}\sum_{i\in I,t\in T}\;AI_{\mathrm{it}}U_{\mathrm{it}}$ follows a normal distribution with a mean of zero. However, for ${\left(\frac{1}{n}\sum_{i\in I,t\in T}\;A{I_{\mathrm{it}}}^{2}\right)}^{-1}\frac{1}{\sqrt{n}}\sum_{i\in I,t\in T}\;AI_{\mathrm{it}}[g(X_{\mathrm{it}})-\hat{g}(X_{\mathrm{it}})]$ , it is necessary to introduce a regularization term when estimating the specific functional form of $g(X_{\mathrm{it}})$ using machine learning algorithms. While this helps avoid excessive variance in $\hat{g}(X_{\mathrm{it}})$ , it also results in a lack of unbiasedness. Consequently, $\theta_{0}$ struggles to converge to ${\hat{\theta }}_{0}$ .

To accelerate the convergence of $\;\theta_{0}$ towards $\;{\hat{\theta }}_{0}$ , this paper constructs an auxiliary regression:


$$\begin{array}{c} AI_{\mathrm{it}}=m(X_{\mathrm{it}})+V_{\mathrm{it}} \end{array}$$ (5)

$$\begin{array}{c} E(V_{\mathrm{it}}X_{\mathrm{it}})=0 \end{array}$$ (6)

Here, $m(X_{\mathrm{it}})$ represents the regression function of the policy variable on the high-dimensional control variables, and its specific form $\hat{m}(X_{\mathrm{it}})$ also needs to be estimated using machine learning algorithms. $V_{\mathrm{it}}$ denotes the error term, with a conditional mean of zero.

The specific procedure is as follows: First, we estimate $\hat{m}(X_{\mathrm{it}})$ using machine learning algorithms and obtain the residuals denoted as ${\hat{V}}_{\mathrm{it}}=AI_{\mathrm{it}}-\hat{m}(X_{\mathrm{it}})$ . Next, we estimate $\hat{g}(X_{\mathrm{it}})$ using machine learning algorithms, transforming the main regression into $EI_{\mathrm{it}}-\hat{g}(X_{\mathrm{it}})=\theta_{0}AI_{\mathrm{it}}+U_{\mathrm{it}}$ . Finally, we treat $\;{\hat{V}}_{\mathrm{it}}$ as an instrumental variable for $AI_{\mathrm{it}}$ in regression, allowing for the acquisition of unbiased coefficient estimates.


$$\begin{array}{c} {\hat{\theta }}_{0}={\left(\frac{1}{n}\sum_{i\in I,t\in T}{\hat{V}}_{\mathrm{it}}AI_{\mathrm{it}}\right)}^{-1}\frac{1}{n}\sum_{i\in I,t\in T}{\hat{V}}_{\mathrm{it}}[EI_{\mathrm{it}}-\hat{g}(X_{\mathrm{it}})] \end{array}$$ (7)

Through the aforementioned steps, this paper can still obtain an unbiased estimate of treatment effects using Double Machine Learning, even in the context of unknown functional forms of covariates. Moreover, the impact of artificial intelligence development on urban pollutant and carbon emission reduction exhibits heterogeneous effects across different geographical locations and cities with varying characteristics. To account for these heterogeneous characteristics in the model estimation, this paper establishes a more general interaction model (38):


$$\begin{array}{c} EI_{\mathrm{it}}=g(AI_{\mathrm{it}},X_{\mathrm{it}}\;)+U_{\mathrm{it}},E(U_{\mathrm{it}}|X_{\mathrm{it}},AI_{\mathrm{it}})=0 \end{array}$$ (8)

$$\begin{array}{c} AI_{\mathrm{it}}=m(X_{\mathrm{it}})+V_{\mathrm{it}}E(V_{\mathrm{it}}|X_{\mathrm{it}})=0 \end{array}$$ (9)

The estimation methods for the relevant parameters are consistent with those of the partially linear model.

#### 3.1.2 Indicator construction

##### 3.1.2.1 Core dependent variable

Pollutant and Carbon Emission Reduction Index (EI). It represents the degree of reverse pollutant and carbon emission reduction; the larger the index, the higher the pollution level and the lower the degree of pollutant and carbon emission reduction. This variable is assessed comprehensively by combining CO2 emissions and PM2.5 concentration using the entropy weighting method. First, based on environmental protection and sustainable development policies, CO2 emissions and PM2.5 concentration are regarded as important adverse indicators, as these two indicators directly reflect a city’s effectiveness and pressure in pollutant and carbon emission reduction. Simultaneously, panel data from relevant cities in China are utilized to calculate the normalized values of each indicator, eliminating the influence of different units and magnitudes. Next, the entropy weighting method is applied to weight and synthesize these two adverse indicators (39). First, the entropy value of each indicator is calculated to assess its informational contribution to pollutant and carbon emission reduction. Subsequently, the degree of divergence is calculated based on the entropy values, ultimately determining the weights for each adverse indicator.

The specific steps are as follows:

For CO2 emissions $\left(\mathrm{CO}2_{i}\right)$ and PM2.5 concentration $\left(\mathrm{PM}2.5_{i}\right)$ , we first standardize these two indicators:


$$\begin{array}{c} x_{\mathrm{CO}2_{i}}=\frac{\mathrm{CO}2_{i}-r_{\min}}{r_{\max}-r_{\min}} \end{array}$$ (10)

$$\begin{array}{c} x_{\mathrm{PM}2.5_{i}}=\frac{\mathrm{PM}2.5_{i}-r_{\min}}{r_{\max}-r_{\min}} \end{array}$$ (11)

Here, $r_{\max}$ and $\;r_{\min}$ represent the maximum and minimum values of the indicator among all observations, respectively. For cases where the standardized result is 0, we set its value to 0.00001 to avoid division by zero errors.

(2) Calculate the weight of each indicator:


$$\begin{array}{c} \mathrm{sum}(x_{i})=x_{\mathrm{CO}2_{i}}+x_{\mathrm{PM}2.5_{i}} \end{array}$$ (12)

After summing the standardized values of all cities, calculate the weight of each indicator:


$$\begin{array}{c} y_{\mathrm{CO}2_{i}}=\frac{x_{\mathrm{CO}2_{i}}}{\mathrm{sum}(x)} \end{array}$$ (13)

$$\begin{array}{c} y_{\mathrm{PM}2.5_{i}}=\frac{x_{\mathrm{PM}2.5_{i}}}{\mathrm{sum}(x)} \end{array}$$ (14)

(3) Calculate the entropy value of each indicator:


$$\begin{array}{c} E_{\mathrm{CO}2}=-\frac{1}{\ln(N)}\sum_{j=1}^{N}y_{\mathrm{CO}2_{j}}\ln(y_{\mathrm{CO}2_{j}}) \end{array}$$ (15)

$$\begin{array}{c} E_{\mathrm{PM}2.5}=-\frac{1}{\ln(N)}\sum_{j=1}^{N}y_{\mathrm{PM}2.5_{j}}\ln(y_{\mathrm{PM}2.5_{j}}) \end{array}$$ (16)

Where (*N*) is the sample size.

(4) Calculate the degree of divergence:


$$\begin{array}{c} d_{\mathrm{CO}2}=1-E_{\mathrm{CO}2}d_{\mathrm{PM}2.5}=1-E_{\mathrm{PM}2.5} \end{array}$$ (17)

(5) Calculate the weight of each indicator:


$$\begin{array}{c} W_{\mathrm{CO}2}=\frac{d_{\mathrm{CO}2}}{d_{\mathrm{CO}2}+d_{\mathrm{PM}2.5}} \end{array}$$ (18)

$$\begin{array}{c} W_{\mathrm{PM}2.5}=\frac{d_{\mathrm{PM}2.5}}{d_{\mathrm{CO}2}+d_{\mathrm{PM}2.5}} \end{array}$$ (19)

(6) Calculate the Comprehensive Pollution Reduction and Carbon Reduction Index (EI):


$$\begin{array}{c} \mathrm{EI}=W_{\mathrm{CO}2}\cdot x_{\mathrm{CO}2}+W_{\mathrm{PM}2.5}\cdot x_{\mathrm{PM}2.5} \end{array}$$ (20)

The Pollutant and Carbon Emission Reduction Index (EI) comprehensively considers the impacts of two adverse indicators: CO2 emissions and PM2.5 concentration. As this index increases, it indicates a rise in pollution levels; conversely, a decrease implies a reduction in pollution levels and an improvement in pollution reduction and carbon emission performance.

##### 3.1.2.2 Core explanatory variables

The development of artificial intelligence (AI) is represented by the density of industrial robot installations in prefecture-level cities. Industrial robots, as an important carrier of AI technology in industrial applications, possess an installation density that effectively reflects the penetration of AI in industrial production and related sectors, thereby indicating the potential implications of AI development for pollutant and carbon emission reduction.

##### 3.1.2.3 Mechanism variables

To investigate the mechanism of the impact of artificial intelligence development on pollutant and carbon emission reduction, this paper aims to reveal its mechanistic effects through three pathways: the optimization effect of green total factor energy efficiency, the reverse pressure effect of industrial structure upgrading, and the driving effect of green technology innovation.

Green Total Factor Energy Efficiency (Gtfe). This paper employs the Slack-Based Measurement model to measure green total factor energy efficiency. The SBM model adequately considers energy inputs and undesirable outputs, providing a more precise reflection of efficiency during the energy utilization process. Higher green total factor energy efficiency indicates more efficient energy utilization in production and related activities, leading to relatively lower pollution emissions and contributing to pollutant and carbon emission reduction.

Industrial Structure (Ins). The proportion of the tertiary sector is an important indicator for measuring industrial structure. The tertiary sector typically exhibits relatively low energy consumption and pollution emissions. An increase in the proportion of the tertiary sector, indicating an upgrade to a more advanced industrial structure, exerts pressure on high energy-consuming and high-polluting industries, promoting a transition in economic development modes and consequently having a positive impact on pollutant and carbon emission reduction. This paper measures the proportion of the tertiary sector using the share of tertiary sector value added to regional GDP in prefecture-level cities.

Green Technology Innovation (Lngrva). The number of green invention patent applications serves as a direct measure of green technology innovation activities. A greater number of green invention patent applications implies that cities are making more investments and yielding higher outputs in green technology research and development. These green technologies can be applied in production, pollution control, and other processes, driving the progress of pollutant and carbon emission reduction. This paper measures the level of green technology innovation using the logarithm of the number of green invention patent applications in prefecture-level cities.

##### 3.1.2.4 Control variables

By relying on the double machine learning method, we can effectively address the challenges associated with high-dimensional control variables, thereby enhancing the accuracy and robustness of the estimates. Building on existing relevant research, this paper incorporates additional factors that may influence pollutant and carbon emission reduction as control variables, as follows:

Economic Development Level (Pgdp), measured by per capita regional GDP;

Financial Development Level (Fin), assessed using the ratio of total deposits and loans of financial institutions to regional GDP;

Level of Openness (Open), represented by the proportion of actual utilized foreign capital to regional GDP;

Urbanization Rate (Urban), measured by the ratio of urban population to total population;

Government Intervention (Gov), measured by the proportion of local government general budget expenditure to regional GDP;

Fiscal Investment (Fiscal), indicated by the ratio of total local government investment to regional GDP;

Upgrading of Industrial Structure (Ind), represented by the ratio of value added in the tertiary sector to that in the secondary sector;

Level of Human Capital (Hc), measured by the number of individuals with college degrees or higher per 10,000 people.

To control for innate characteristics at the city and time levels, city fixed effects and time fixed effects are included in the regression analysis.

#### 3.1.3 Data sources

This paper utilizes panel data from 282 prefecture-level and above cities in China covering the years 2009 to 2023, with the data sources outlined as follows: Firstly, the core variable data primarily originates from publicly available authoritative statistical yearbooks such as the “China Urban Statistical Yearbook,” “China Environmental Statistical Yearbook,” “China Science and Technology Statistical Yearbook,” and “China Energy Statistical Yearbook.” The data on industrial robot installation density is supplemented with industrial development reports and relevant white papers published by the industrial and information departments of each city. The CO₂ emission data required for the pollutant and carbon emission reduction index is sourced from relevant databases of the Global Carbon Project and carbon emission inventories published by provincial ecological and environmental departments. The PM₂.₅ concentration data is compiled using information from the China Air Quality Online Monitoring Analysis Platform, in conjunction with publicly available data from urban environmental monitoring stations. Input–output data necessary for measuring green total factor energy efficiency is derived from the aforementioned statistical yearbooks and municipal statistical bulletins. Secondly, the tool variable data for “the minimum distance between prefecture-level cities and backbone optical cable cities” is obtained from the national geographic information public service platform released by the National Administration of Surveying, Mapping and Geo-information, which provides geographic coordinate information for the cities. The list of backbone optical cable cities is determined based on the “National Backbone Optical Cable Network Construction Planning” released by the Ministry of Industry and Information Technology and related reports in the telecommunications industry. Geographic Information System (GIS) is then utilized to calculate the minimum distance between each city and the backbone optical cable cities, while cross-validation is performed using publicly available network layout information from telecommunications operators. Thirdly, the data on green invention patent applications is sourced from the Patent Information System of the National Intellectual Property Administration. Data on the proportion of the tertiary sector and other aspects of industrial structure, as well as financial development level data, are extracted from the “China Urban Statistical Yearbook” and “China Financial Statistical Yearbook,” ensuring the accuracy and authority of the data (Table 1).

**Table 1:** Descriptive statistics of variables.

Var	Obs	Mean	p50	SD	Min	Max
EI	4,200	0.599	0.639	0.203	0.060	1
AI	4,200	5.035	4.832	1.951	0.693	11.54
Pgdp	4,200	10.72	10.65	0.733	8.391	13.19
Fin	4,200	2.563	2.257	1.245	0.588	21.30
Open	4,200	0.002	0.002	0.003	0	0.029
Urban	4,200	0.395	0.339	0.211	0.075	1
Gov	4,200	0.197	0.171	0.100	0.044	1.027
Fiscal	4,200	5.884	4.905	4.181	0.010	41.68
Ind	4,200	1.071	0.933	0.615	0.109	6.383
Hc	4,200	0.020	0.011	0.025	0	0.185
Gtfe	4,200	0.344	0.316	0.142	0.103	1.193
Lngrva	4,200	3.993	3.807	1.775	0	10.08
Ins	4,200	0.426	0.423	0.103	0.098	0.848
IV	4,200	126.5	122.5	93.23	0	604.5

### 3.2 Benchmark analysis

This paper employs a double machine learning method to assess the impact of artificial intelligence development on urban pollutant and carbon emission reduction. In the study, the sample is split in a 1:4 ratio, and the random forest algorithm is utilized to fit both the main regression and the auxiliary regression, with the primary regression results presented in Table 2. Column (1) controls for city and time fixed effects, as well as the first-order terms of various city characteristics across the full sample. The results indicate that the regression coefficient for the impact of artificial intelligence development on urban pollutant and carbon emission reduction is negative and significant at the 1% level, suggesting that the development of artificial intelligence significantly reduces urban pollutant and carbon emission reduction.

**Table 2:** Benchmark regression results.

Var	(1)	(2)	(3)	(4)
	EI	EI	EI	EI
*AI*	−0.0260^***^	−0.0260^***^	−0.0160^***^	−0.0162^***^
	(0.0024)	(0.0024)	(0.0029)	(0.0029)
*Constant*	−0.0023^*^	−0.0023^*^	−0.0032^**^	−0.0032^*^
	(0.0013)	(0.0013)	(0.0016)	(0.0016)
*Control*	Yes	Yes	Yes	Yes
*Control^2^*	No	Yes	No	Yes
*City FE*	Yes	Yes	Yes	Yes
*Time FE*	Yes	Yes	Yes	Yes
*Dml*	RF	RF	RF	RF
*Obs*	4,200	4,200	3,097	3,097

Standard errors are in parentheses, ***, **, *represent 1, 5, and 10%, respectively.

Building on this, Column (2) further incorporates the quadratic terms of city variables. The results remain significantly positive, with coefficients displaying minimal variation. Additionally, Column (3) and (4) restrict the sample period to 2012–2022 and conduct the regression analysis again. The results show that, despite a decrease in the absolute value of the regression coefficient for artificial intelligence development after shortening the sample period, its impact on urban pollutant and carbon emission reduction remains significant, confirming the validity of Hypothesis 1.

### 3.3 Robustness analysis

#### 3.3.1 Adjust the research sample

In light of the significant regional heterogeneity among prefecture-level cities in China regarding the foundation of the artificial intelligence industry, energy consumption structure, and environmental governance capacity, the municipalities directly under the central government (Beijing, Shanghai, Tianjin, and Chongqing), as national-level central cities, exhibit a far higher installation density of industrial robots and investment in green technology research and development compared to ordinary prefecture-level cities. Furthermore, they possess independent policy authority in areas such as pollutant and carbon emission reduction pilot programs and fiscal resource allocation, which creates distinct stratification differences in core variable characteristics and policy environments compared to other prefecture-level cities. Including these municipalities in the sample could potentially lead to biased estimation results.

Based on this, this paper excludes the aforementioned four municipalities in the empirical analysis, retaining the remaining 278 prefecture-level cities as the research sample to avoid sample selection bias caused by special administrative levels, thereby enhancing the representativeness of regression results for ordinary prefecture-level cities and the accuracy of core causal effect estimates. The results are presented in Table 3 (1). The regression results indicate that after excluding the municipalities directly under the central government, the impact coefficient of artificial intelligence development on urban pollutant and carbon emission reduction exhibits some variation; however, its positive effect remains significant at the 1% level. This indicates that the baseline regression results exhibit strong robustness.

**Table 3:** Robustness testI.

Var	(1)	(2)	(3)
	EI	EI	EI
*AI*	−0.0247^***^	−0.0259^***^	−0.0248^***^
	(0.0024)	(0.0024)	(0.0023)
*Constant*	−0.0016	−0.0020	−0.0020
	(0.0013)	(0.0013)	(0.0013)
*Control*	Yes	Yes	Yes
*Control^2^*	Yes	Yes	Yes
*City FE*	Yes	Yes	Yes
*Time FE*	Yes	Yes	Yes
*Dml*	RF	RF	RF
*Obs*	4,140	4,200	4,200

Standard errors are in parentheses, ***, **, *represent 1, 5, and 10%, respectively.

#### 3.3.2 Exclude the impact of outliers

Considering that outliers in the regression sample may cause instability in the regression coefficients, resulting in deviations from the true relationships, this paper thus applies 1 and 5% winsorization to all variables, with the regression results presented in Columns (2) and (3) of Table 3. Column (2) shows the regression results after 1% winsorization, while Column (3) presents the results after 5% winsorization. It can be observed that after the removal of outliers, although the regression coefficients exhibit a slight decrease, they remain significant, further corroborating the robustness of the conclusions presented in this paper.

#### 3.3.3 Reset double machine learning model

To eliminate potential biases introduced by the specification of the double machine learning model, this paper conducts robustness checks on the baseline regression results from multiple perspectives: (1) adjusting the division ratio of the training and testing sets from the original 1:4 to 1:2 and 1:7, examining the impact of the division method on the results; (2) replacing the original random forest prediction algorithm with lasso regression, gradient boosting, and support vector machines to compare the effects of different machine learning algorithms on the research conclusions.

Columns (1) to (5) of Table 4 present the regression results after redefining the double machine learning model. The results indicate that regardless of adjustments made to the sample split ratio or the selected machine learning algorithm, the effect of artificial intelligence development on urban pollutant and carbon emission reduction remains significant. However, the estimated values of the policy effects are influenced. These findings indicate that the research conclusions exhibit robustness under different model specifications, thereby further validating the reliability of the baseline regression results.

**Table 4:** Robustness test II.

Var	(1)	(2)	(3)	(4)	(5)	(6)
	Kfolds = 3	Kfolds = 8	Lassocv	Gradboost	Svm	IV
*AI*	−0.0266^***^	−0.0228^***^	−0.0172^***^	−0.0424^***^	−0.0319^***^	−0.3748^***^
	(0.0024)	(0.0023)	(0.0049)	(0.0028)	(0.0024)	(0.1139)
*Constant*	−0.0021	−0.0016	0.0003	−0.0007	−0.0157^***^	−0.0032
	(0.0015)	(0.0013)	(0.0007)	(0.0021)	(0.0028)	(0.0035)
*Control*	Yes	Yes	Yes	Yes	Yes	Yes
*Control* ^*2*^	Yes	Yes	Yes	Yes	Yes	Yes
*City FE*	Yes	Yes	Yes	Yes	Yes	Yes
*Time FE*	Yes	Yes	Yes	Yes	Yes	Yes
*Dml*	RF	RF	*Lassocv*	*Gradboost*	*Svm*	RF
*Obs*	4,200	4,200	4,200	4,200	4,200	4,200

Standard errors are in parentheses, ***, **, *represent 1, 5, and 10%, respectively.

#### 3.3.4 Endogeneity test

The distribution of artificial intelligence development levels also exhibits a degree of non-randomness, which is often influenced by various resource endowment factors, such as the thickness of the regional industrial base, the clustering degree of the manufacturing industry, the intensity of technological research and development investments, and the completeness of high-end equipment supply chains. Cities with a strong manufacturing base are more likely to attract industrial robots, and such cities also have significantly better foundations for pollutant and carbon emission reduction compared to other cities. This may introduce endogeneity issues due to “selection bias,” thereby interfering with the precise identification of the causal effects between artificial intelligence and pollutant and carbon emission reduction.

Based on this, this paper draws on the instrumental variable design approach of double machine learning proposed by Chernozhukov et al. (40), and constructs a partially linear instrumental variable model using double machine learning, aiming to achieve an unbiased estimate of the causal effect of artificial intelligence on pollutant and carbon emission reduction by isolating the endogenous part of the core explanatory variable.


$$\begin{array}{c} EI_{\mathrm{it}}=\theta_{0}AI_{\mathrm{it}}+g(X_{\mathrm{it}})+U_{\mathrm{it}} \end{array}$$ (21)

$$\begin{array}{c} IV_{\mathrm{it}}=m(X_{\mathrm{it}})+V_{\mathrm{it}} \end{array}$$ (22)

Here, $IV_{\mathrm{it}}$ represents the instrumental variable. In this study, the minimum distance (unit: km) between prefectural-level cities and optical cable backbone cities is employed as the instrumental variable corresponding to the core explanatory variable, namely the installation density of industrial robots. On one hand, the minimum distance is jointly determined by the geographical locations of the cities and the layout of the national optical cable backbone network, which serves as a geographic locational characteristic variable. The location of optical cable backbone cities is primarily based on the overall planning of the national communication network and has no direct correlation with the pollutant and carbon emission reduction levels of individual prefectural-level cities or their demand for industrial robot applications. Furthermore, the geographical distance is not affected by endogenously reverse effects from urban economic activities, environmental governance policies, or similar factors, thereby strictly meeting the exogeneity assumption of instrumental variables.

On the other hand, optical cable backbone cities serve as core hubs for regional digital information transmission, and the distance to these cities directly affects the accessibility of digital infrastructure in prefectural-level cities: the closer the distance, the more developed the digital infrastructure, which is conducive to the networking operation, data interaction, and intelligent application of industrial robots, thereby significantly affecting the installation density of industrial robots. Meanwhile, this geographical distance itself does not directly affect the pollutant and carbon emission reduction index, but rather indirectly impacts the pollutant and carbon emission reduction processes by influencing the installation density of industrial robots, fully satisfying the relevance and exclusion assumptions of instrumental variables.

Column (6) of Table 4 indicates that the estimated coefficient of the core explanatory variable, artificial intelligence development, remains significantly negative for the dependent variable, the pollutant and carbon emission reduction index, which is consistent with the direction of the baseline regression results, thus validating the effectiveness of the instrumental variable model estimates.

### 3.4 Mechanism analysis

This study identifies green total factor energy efficiency, industrial structure optimization, and green technology innovation as the three core mechanisms through which artificial intelligence (AI) drives pollution reduction and carbon abatement. Although potential interconnections exist among these three factors, there is ample justification for treating them as independent variables in the research, as elaborated below: First, green total factor energy efficiency measures the comprehensive resource and environmental performance of an economic system, directly reflecting the balance between economic output, pollutant emissions, and given energy inputs. It can independently capture the direct effects of AI in optimizing resource allocation and reducing energy consumption. Second, industrial structure optimization focuses on the low-carbon transformation of the economy. Its changes are jointly driven by policy guidance and market forces, forming a relatively independent impact chain. Moreover, industrial structure optimization involves the restructuring of different economic sectors, and its relationship with energy efficiency is non-linear and complex, which necessitates its independent analysis. Third, green technology innovation is a key driver of sustainable development. This study focuses on AI-empowered green technology innovation practices—while such innovation may improve energy efficiency, its core value lies in unlocking new possibilities for urban pollution reduction and carbon abatement, underscoring the significance of its independent investigation. From a policy practice perspective, policymakers need to formulate targeted measures based on the independent role of each mechanism. Treating the three mechanisms as independent variables not only clarifies the distinct pathways through which AI facilitates pollution reduction and carbon abatement, but also enhances the rigor of the theoretical framework and the reliability of empirical analysis.

#### 3.4.1 Green total factor energy efficiency

Artificial intelligence influences urban pollutant and carbon emission reduction by optimizing energy utilization efficiency, thereby reducing pollution and carbon emissions per unit of output and ensuring the synergistic optimization of economic output, energy savings, and pollution reduction. Specifically, industrial robots collect energy consumption and emission data in real time, dynamically adjust production parameters, and plan energy consumption timings in advance, thereby reducing energy waste and redundancy, improving efficiency, and subsequently promoting urban pollutant and carbon emission reduction.

At the same time, green total energy efficiency is measured using the SBM-DEA model, with fixed asset investment, the number of employees, and total energy consumption as input variables, and regional GDP as the expected output variable. Industrial sulfur dioxide emissions, industrial nitrogen oxides emissions, and carbon dioxide emissions serve as non-desired output variables. This approach utilizes a non-radial and non-angular measurement method to avoid the shortcomings of traditional efficiency measurement that overlook non-desired outputs.

Column (1) of Table 5 shows the impact of artificial intelligence development on green total energy efficiency. It can be observed that the development of artificial intelligence significantly promotes the improvement of green total energy efficiency, and this effect remains significant at the 1% level after controlling for variables such as the level of economic development, financial development, and urbanization rate. This indicates that the development of artificial intelligence indirectly drives the achievement of pollutant and carbon emission reduction goals through this mechanism, thereby validating the mediating role of green total energy efficiency in the relationship between artificial intelligence development and pollutant and carbon emission reduction, thus confirming the hypothesis.

**Table 5:** Mechanism analysis.

Var	(1)	(2)	(3)
	Gtfe	Ins	Lngrva
*AI*	0.0207^***^	0.0017^***^	0.5235^***^
	(0.0025)	(0.0005)	(0.0170)
*Constant*	0.0025^**^	−0.0003	−0.0096
	(0.0011)	(0.0003)	(0.0090)
*Control*	Yes	Yes	Yes
*Control^2^*	Yes	Yes	Yes
*City FE*	Yes	Yes	Yes
*Time FE*	Yes	Yes	Yes
*Dml*	RF	RF	RF
*Obs*	4,200	4,200	4,200

Standard errors are in parentheses, ***, **, *represent 1, 5, and 10%, respectively.

#### 3.4.2 Industrial structure

One of the core mechanisms through which the development of artificial intelligence impacts urban pollutant and carbon emission reduction is by promoting industrial structure optimization and reducing the proportion of high-energy-consuming industries. Industrial structure optimization primarily manifests in the transition from the secondary sector to the tertiary sector, as well as the upgrading of high-energy-consuming industries to low-energy-consuming industries. This transformation effectively reduces pollutant emissions and carbon emissions.

In this process, artificial intelligence achieves the goals of pollutant and carbon emission reduction through three aspects of empowerment: First, industrial robots replace low-end, high-energy-consuming jobs, prompting a shift of labor towards the low-energy-consuming tertiary sector, thereby reducing overall unit energy consumption and pollutant emissions. Second, intelligent production technologies drive structural upgrading within the secondary sector by phasing out high-energy-consuming outdated capacities and developing low-energy-consuming industries, thus achieving reductions in carbon emissions. Finally, digital platforms optimize the allocation of industrial resources, reducing dependency on high-energy-consuming industries and guiding resources to concentrate in low-emission areas, effectively lowering overall pollution emissions and carbon footprints.

Column (2) of Table 5 shows the impact of artificial intelligence development on industrial structure. It can be observed that the improvement in artificial intelligence levels significantly promotes the increase in the share of the tertiary sector in total output, and this effect remains significant at the 1% level after controlling for variables such as economic development level, financial development level, and urbanization rate. With the optimization of industrial structure, the overall energy consumption intensity in the region also decreases. The development of artificial intelligence indirectly drives urban pollutant and carbon emission reduction through this mechanism, thereby validating the mediating role of industrial structure in the relationship between artificial intelligence development and urban pollutant and carbon emission reduction, thus confirming the hypothesis.

#### 3.4.3 Green technology innovation

Another core mechanism through which artificial intelligence promotes pollutant and carbon emission reduction is by facilitating green technology innovation, thereby reducing pollution and carbon emissions per unit of output. Green technology innovation can reduce pollutant emission intensity at the source, enhancing pollutant treatment efficiency through advanced governance technologies and leveraging low-carbon technologies to decrease carbon emissions in production processes, ultimately achieving the synergistic advancement of pollutant and carbon emission reduction.

The development of artificial intelligence drives green technology innovation and assists in emission reductions through three aspects of technological empowerment. First, data accumulated from production and emissions by industrial robots provides empirical evidence for green technology research and development, enabling precise identification of directions for technological breakthroughs. Second, machine learning algorithms accelerate the research and development process, shortening the time it takes for green technologies to transition from laboratories to industrialization. Finally, practical applications of green technologies are promoted through features such as intelligent monitoring and automated control, broadening the applicability of technologies in high-energy-consuming industries, thereby enhancing the effectiveness of green technology innovation and advancing urban pollutant and carbon emission reduction.

Column (3) of Table 5 presents the impact of artificial intelligence development on green technology innovation. It can be observed that advancements in artificial intelligence significantly promote an increase in the number of green invention patent applications, and this effect remains significant at the 1% level even after controlling for a series of foundational variables. As the capability for green technology innovation improves, regional industrial pollution treatment efficiency and low-carbon production levels increase concurrently. Through this mechanism, artificial intelligence indirectly drives the achievement of pollutant and carbon emission reduction goals, thereby validating the mediating role of green technology innovation in the relationship between artificial intelligence development and pollutant and carbon emission reduction, thus confirming the hypothesis.

## 4 Further analysis

### 4.1 Heterogeneity of urban clusters

The differences in industrial foundations, the penetration of digital technologies, and environmental governance endowments among regions in China have led to significant spatial heterogeneity in the impact of artificial intelligence on pollutant and carbon emission reduction. This study categorizes the sample according to the “China Urban Agglomeration Development Report,” dividing it into five major urban agglomerations: Beijing-Tianjin-Hebei, the Yangtze River Delta, the Central Yangtze River, Chengdu-Chongqing, and the Pearl River Delta, while establishing a control group of non-urban agglomerations to investigate the differences in the impact of artificial intelligence on pollution reduction and carbon emission cuts across these urban groups.

According to the regression results in Table 6, the development of artificial intelligence has a significant promoting effect on pollutant and carbon emission reduction in the Beijing-Tianjin-Hebei, Chengdu-Chongqing, and non-urban agglomeration areas, while its effect on the Yangtze River Delta, Central Yangtze River, and Pearl River Delta urban agglomerations is not significant.

**Table 6:** Heterogeneity analysis I: Heterogeneity of urban agglomerations.

Var	(1)	(2)	(3)	(4)	(5)	(6)
	Beijing-Tianjin-Hebei	Yangtze River Delta	Middle Reaches of the Yangtze	Chengdu-Chongqing	Pearl River Delta	Non-urban Clusters
*AI*	−0.0166^***^	0.0008	−0.0067	−0.0082^*^	−0.0041	−0.0164^***^
	(0.0061)	(0.0046)	(0.0052)	(0.0042)	(0.0051)	(0.0024)
*Constant*	0.0030	−0.0032	−0.0026	−0.0005	−0.0012	−0.0018
	(0.0037)	(0.0022)	(0.0023)	(0.0028)	(0.0027)	(0.0014)
*Control*	Yes	Yes	Yes	Yes	Yes	Yes
*Control^2^*	Yes	Yes	Yes	Yes	Yes	Yes
*City FE*	Yes	Yes	Yes	Yes	Yes	Yes
*Time FE*	Yes	Yes	Yes	Yes	Yes	Yes
*Dml*	RF	RF	RF	RF	RF	RF
*Obs*	210	390	419	224	135	2,822

Standard errors are in parentheses, ***, **, *represent 1, 5, and 10%, respectively.

Specifically, the industrial concentration in the Beijing-Tianjin-Hebei region is high, and artificial intelligence significantly improves pollutant and carbon emission reduction by optimizing production processes. The Chengdu-Chongqing region is primarily focused on automotive manufacturing and electronic assembly, where the application of artificial intelligence emphasizes efficiency improvements and emissions reduction, resulting in outstanding performance in pollutant and carbon emission reduction.

The low industrial technology level in non-urban agglomerations enhances the adaptability of artificial intelligence, enabling it to quickly elevate the effects of pollutant and carbon emission reduction. In contrast, the industrial modernization level in the Yangtze River Delta is high; however, the core demand is gradually shifting towards breakthroughs in green technologies, resulting in diminishing marginal returns for artificial intelligence.

In the Central Yangtze River region, limitations in industrial structure and technological application yield insignificant emission reduction effects, while the Pearl River Delta region, primarily consisting of light industry and high-tech industries, faces insufficient significance in emission reduction increments, leading to a lack of noticeable effects on pollutant and carbon emission reduction.

### 4.2 Heterogeneity of cities in the eight major economic zones

From Table 7, it is evident that there are significant discrepancies in the impact of artificial intelligence development on pollutant and carbon emission reduction across the eight major comprehensive economic zones in China. The effects in the northern coastal region, the middle reach of the Yellow River, the southwestern region, and the northwestern region all have passed significance testing. In contrast, the coefficients for the northeastern region, eastern coastal region, southern coastal region, and the middle reach of the Yangtze River do not pass significance testing, revealing a distinct distribution of significant regional effects overall.

**Table 7:** Heterogeneity analysis III: Heterogeneity of the eight comprehensive economic zones.

Var	(1)	(2)	(3)	(4)	(5)	(6)	(7)	(8)
	Northeast	Northern Coastal Areas	Eastern Coastal Areas	Southern Coastal Areas	Middle Reaches of the Yellow	Middle Reaches of the Yangtze River	Southwest	Northwest
*AI*	−0.0080	−0.0117^***^	0.0104	0.0026	−0.0368^***^	−0.0008	−0.0349^***^	−0.0140^***^
	(0.0053)	(0.0045)	(0.0066)	(0.0028)	(0.0056)	(0.0040)	(0.0037)	(0.0045)
*Constant*	0.0013	0.0038^*^	−0.0026	−0.0003	0.0007	−0.0001	−0.0002	−0.0013
	(0.0020)	(0.0022)	(0.0024)	(0.0013)	(0.0028)	(0.0018)	(0.0021)	(0.0027)
*Control*	Yes	Yes	Yes	Yes	Yes	Yes	Yes	Yes
*Control* ^*2*^	Yes	Yes	Yes	Yes	Yes	Yes	Yes	Yes
*City FE*	Yes	Yes	Yes	Yes	Yes	Yes	Yes	Yes
*Time FE*	Yes	Yes	Yes	Yes	Yes	Yes	Yes	Yes
*Dml*	RF	RF	RF	RF	RF	RF	RF	RF
*Obs*	489	435	375	480	699	779	649	294

Standard errors are in parentheses, ***, **, *represent 1, 5, and 10%, respectively.

In terms of the relationship between regional actual conditions and the adaptability of artificial intelligence applications, the regions with significant effects have formed strong emission reduction support systems based on their foundational conditions. The northern coastal region relies on the characteristics of its industrial system and digital infrastructure, allowing industrial robots to play a substantial role in emission reductions within high-energy-consuming industries. The middle reach of the Yellow River, as a concentration area for energy industries, aligns artificial intelligence technologies directly with the pollutant and carbon emission reduction needs of traditional energy sectors. The southwestern region leverages its clean energy advantages to integrate artificial intelligence with green production in specialized industries, thereby fostering collaborative efforts for emission reductions. The northwestern region gradually enhances its application of industrial robots by utilizing renewable energy and developing digital infrastructure, aiming to address technological deficits in emission reduction.

Conversely, the regions with insignificant effects are constrained by various real-world conditions. In the northeastern region, the integration of industrial applications and investments has yet to form effective connections, hindering the release of effects. The eastern coastal region faces a contraction of traditional emission reduction opportunities, with a disparity between current demands for pollutant and carbon emission reduction and the existing directions of artificial intelligence applications. The southern coastal region exhibits limited emission reduction potential due to a low baseline of industrial emissions. Finally, in the middle reach of the Yangtze River, the application of artificial intelligence lacks comprehensive process coordination, and uneven policy implementation has prevented effects from fully materializing.

## 5 Research conclusions and policy recommendations

### 5.1 Conclusion

In the urgent timeframe for addressing global challenges of pollutant and carbon emission reduction, exploring the impact of artificial intelligence development on urban pollutant and carbon emission reduction, especially its effective role in advancing urban transformation and green technology development, carries significant practical implications. Meanwhile, the improvement of urban environmental quality is directly tied to the promotion of residents’ health and well-being, and this research perspective is also consistent with the core concerns of the public health field. Utilizing panel data from 282 prefecture-level cities in China spanning from 2009 to 2023, this paper employs a dual machine learning model to elucidate the mechanisms through which artificial intelligence development contributes to pollutant and carbon emission reduction.

The study finds that (1) the development of artificial intelligence significantly enhances green total factor energy efficiency, optimizes energy utilization; reducing the erosion of urban ecological environment by pollutant and carbon emissions, thereby building a solid environmental barrier for residents’ health, and promotes urban pollutant and carbon emission reduction. (2) Artificial intelligence development facilitates the optimization of industrial structure, leading to a shift of resources toward the low-energy-consumption and low-pollution tertiary sector, reducing the health risks of industrial pollution to densely populated areas, thereby advancing urban pollutant and carbon emission reduction. (3) The growth of artificial intelligence accelerates green technology innovation, increasing the number of green invention patent applications and fundamentally reducing carbon emissions, thus establishing a long-term mechanism for pollutant and carbon emission reduction.

Heterogeneity analysis indicates that the impact of artificial intelligence development on pollutant and carbon emission reduction varies across urban agglomerations, regions, and economic zones: the emission reduction effects are significant in the Beijing-Tianjin-Hebei region, Chengdu-Chongqing, and non-urban agglomerations, and the improvement of environmental quality in these regions can more easily alleviate the high incidence of local diseases such as respiratory and cardiovascular illnesses, while in the Yangtze River Delta, the middle reach of the Yangtze River, and the Pearl River Delta, the effects are limited due to insufficient green technology transformation and efficiency. Moreover, artificial intelligence significantly promotes pollutant and carbon emission reduction in the northern coastal region, the middle reach of the Yellow River, the southwestern region, and the northwestern region, providing technical support for public health protection in these ecologically fragile regions, while the northeastern region, eastern coastal region, southern coastal region, and middle reach of the Yangtze River face challenges related to uneven industrial structure and policy implementation.

### 5.2 Policy recommendations

1. Strengthen policy support for artificial intelligence in environmental protection. The government should increase support for the research, development, and application of artificial intelligence technologies, particularly in investments related to environmental protection. Establishing special funds would incentivize enterprises and research institutions to apply industrial robots and intelligent monitoring systems in high-energy-consuming industries to enhance resource utilization efficiency and reduce pollution emissions, thereby reducing the harm of environmental pollutants to public health at the source. Additionally, moderate fiscal and tax policies, such as tax reductions and subsidies for green technology investments, should be implemented to stimulate enterprises to actively transition towards green technologies, facilitating the coordinated advancement of sustainable development and public health protection.
2. Promote industrial structure transformation. To facilitate high-quality economic development, policies should be formulated that encourage the labor force to transition toward the tertiary sector and reduce dependency on high-pollution industries, especially in regions with weaker foundations—this transformation can not only optimize the economic structure, but also reduce the health risks of residents exposed to industrial pollution. The government should guide resources toward low-pollution and low-energy-consumption industries through subsidies and investments, such as promoting the development of green service industries and renewable energy sectors. Moreover, training and re-education mechanisms should be established to assist workers in making a smooth transition, enhancing their competitiveness in emerging industries.
3. Create a supportive policy environment for green technology innovation. To effectively promote the research and development of green technologies, the government needs to provide financial and technical support, establish collaborative platforms for industry, academia, and research, and encourage joint investments in green technology R&D by research institutions and enterprises. Strengthening mechanisms for the protection and transfer of intellectual property rights is essential to improve the efficiency and conversion rates of technology applications, enabling more green technologies to be implemented effectively and helping to solve public health problems caused by environmental pollution. Furthermore, establishing awards for green technology innovation could incentivize corporate innovation, ensuring green technologies effectively support pollutant and carbon emission reduction.
4. Implement differentiated regional policies. Given the varying levels of economic development and environmental protection capabilities across different regions, the government should adopt tailored emission reduction policies to assist regions with weaker technological foundations in conducting research and applying emission reduction technologies that suit their specific characteristics. These policies could encompass financial support and technical assistance for emerging environmental protection projects, promoting green transformation in these areas, thereby improving the basic guarantee level of regional public health services. Additionally, drawing lessons from successful experiences in regions with advanced technological development would facilitate greater promotion of green technologies and foster nationwide sharing and cooperation, achieving nationwide coordinated development of environmental quality improvement and public health enhancement.

## 6 Research limitations and prospects

Based on the panel data of prefecture-level cities in China from 2009 to 2023, this study uses the double machine learning model to reveal the impact, mechanism and regional heterogeneity of artificial intelligence (AI) on urban pollutant and carbon emission reduction. Despite achieving certain research results, there are still obvious limitations. First, there is a boundary constraint in the measurement of the core explanatory variable. This study uses industrial robot installation density to characterize the development level of AI, which can reflect the application of AI technology in the industrial field, but fails to cover service-end and consumer-end AI technologies such as natural language processing and intelligent algorithm decision-making. As a result, the research conclusions are only applicable to pollutant and carbon emission reduction in the field of industrial automation, and cannot fully present the emission reduction value of full-scenario AI technologies. Second, there are limitations in the depth and dimension of mechanism analysis. Although this study identifies three core transmission paths, namely green total factor energy efficiency, industrial structure optimization and green technology innovation, it does not explore the interaction between these three mechanisms, nor does it distinguish the mechanism differences of AI in different emission reduction stages such as source emission reduction and end-of-pipe treatment, resulting in insufficient refinement of mechanism analysis. Third, the subdivision dimension of regional heterogeneity is insufficient. The study only discusses from the levels of urban agglomerations and eight comprehensive economic zones, and fails to explore the heterogeneity of micro-dimensions such as city size, resource endowment and policy pilots, making it difficult to accurately match the technology adaptation paths of different types of cities.

In view of the above research limitations, combined with the development trend of AI and green low-carbon transformation, future research can expand the analysis scope from multiple dimensions. At the level of variable measurement, multi-source data such as the number of registered AI enterprises, the number of AI technology patents and digital infrastructure coverage can be integrated to construct a comprehensive AI development index. Meanwhile, application data of service-end and consumer-end AI technologies can be included to break through the category limitation of the single indicator of industrial robots and comprehensively investigate the impact of full-scenario AI technologies on pollutant and carbon emission reduction. At the level of mechanism analysis, a mechanism interaction model can be further constructed to explore the synergistic or restrictive relationship among green total factor energy efficiency, industrial structure optimization and green technology innovation. Meanwhile, based on the division of emission reduction links, the mechanism differences of AI in different links such as source emission reduction, process control and end-of-pipe treatment can be analyzed to clarify the precise path of technology-enabled emission reduction.

Future research can also be deepened from the aspects of regional heterogeneity subdivision and method-scenario innovation. In terms of regional heterogeneity, analysis can be carried out from micro-dimensions such as city size, resource endowment and policy attributes, and the differences in AI’s emission reduction effects between resource-based and non-resource-based cities, as well as between “dual carbon” pilot and non-pilot cities can be compared. Combined with spatial analysis methods such as the geographically weighted model, the spatial spillover characteristics of AI’s emission reduction effects can be revealed to provide more accurate basis for formulating differentiated policies. In terms of methods and scenarios, the quantile double machine learning model can be introduced to identify the marginal effect differences of AI in cities with different levels of pollution reduction and carbon abatement. Meanwhile, the synergistic effect of AI with policy tools such as carbon trading markets and energy transformation can be included in the analysis framework to explore the composite emission reduction value of “AI empowering dual carbon goals,” so as to provide more abundant theoretical support and practical reference for realizing the comprehensive green transformation of the economy and society.

## Data Availability

The original contributions presented in the study are included in the article/supplementary material, further inquiries can be directed to the corresponding author.
